# Preoperative Risk Stratification of Adnexal Masses Using the International Ovarian Tumor Analysis (IOTA) Simple Rules and Risk of Malignancy Index (RMI) 2: A Prospective Diagnostic Study From a Tertiary Centre in Southern India

**DOI:** 10.7759/cureus.99699

**Published:** 2025-12-20

**Authors:** Kaarthika T, Sasikala Kathiresan

**Affiliations:** 1 Gynaecology, Sivakasi Nadar Uravinmurai Hospital, Madurai, IND; 2 Obstetrics and Gynaecology, All India Institute of Medical Sciences, Madurai, Madurai, IND

**Keywords:** adnexal mass, diagnostic accuracy, iota simple rules, ovarian cancer, risk of malignancy index 2, ultrasonography

## Abstract

Objective: The objective of this study was to compare the diagnostic accuracy of International Ovarian Tumor Analysis (IOTA) Simple Rules with Risk of Malignancy Index 2 (RMI 2) for preoperative stratification of adnexal masses, and to assess the performance of serum CA-125 and handling inconclusive IOTA results, using histopathology as the reference standard.

Methods: A prospective diagnostic accuracy study was conducted at a tertiary care center in South India, enrolling 56 women aged ≥18 years with ultrasonographically detected adnexal masses scheduled for surgery. IOTA Simple Rules and RMI 2 scoring were applied preoperatively. All patients underwent surgical excision, and the final diagnosis was based on histopathology. Diagnostic metrics, including sensitivity, specificity, positive predictive value (PPV), negative predictive value (NPV), accuracy, and area under the ROC curve (AUC), were calculated. Sensitivity analyses were performed for inconclusive IOTA cases, and statistical comparisons used DeLong and McNemar’s tests.

Results: Of 56 cases, 11 (19.6%) were malignant and 45 (80.4%) benign. IOTA Simple Rules were conclusive in 52 (92.9%) cases. For conclusive IOTA, sensitivity was 90.9%, specificity 95.1%, PPV 83.3%, NPV 97.5%, accuracy 94.2%, and AUC 0.930. RMI 2 showed sensitivity 72.7%, specificity 97.8%, PPV 88.9%, NPV 93.6%, accuracy 92.9%, and AUC 0.853. Sensitivity analyses revealed robust NPV and accuracy with alternative coding of inconclusive IOTA results.

Conclusion: IOTA Simple Rules provide superior sensitivity and comparable specificity compared to RMI 2, supporting their role as a safe and reliable first-line tool for preoperative risk stratification of adnexal masses in gynecologic practice.

## Introduction

Ovarian cancer remains one of the most formidable challenges in gynecologic oncology, accounting for approximately 3% of all cancers among women yet contributing disproportionately to cancer-related mortality due to late-stage diagnosis and subtle early symptoms [1]. Global estimates from the Global Cancer Observatory (GLOBOCAN) 2020 database indicate more than 313,000 new cases and 207,000 deaths annually, with the burden projected to rise particularly in developing countries [2,3]. In India, the incidence of ovarian cancer has shown a steady upward trend, and women frequently present with adnexal masses requiring reliable triage to guide appropriate management. Because early-stage disease is often asymptomatic or manifests with nonspecific complaints, most patients continue to be diagnosed at advanced stages when prognosis is poor and therapeutic options are limited [4].

Accurate preoperative discrimination between benign and malignant adnexal masses is therefore a critical component of clinical care. Timely identification of malignant tumors enables referral to specialized gynecologic oncology centers where optimal surgical staging and cytoreduction can substantially improve survival outcomes [5,6]. Conversely, the majority of adnexal masses are benign and may be managed conservatively or via minimally invasive surgery, helping avoid unnecessary interventions, surgical morbidity, and increased healthcare costs [7]. Effective risk stratification thus supports both improved patient outcomes and more efficient allocation of medical resources.

Several diagnostic tools and risk prediction algorithms have been developed to assist in evaluating adnexal masses. Transvaginal ultrasonography and serum CA-125 measurement form the foundation of initial assessment [8]. The Risk of Malignancy Index (RMI), first introduced by Jacobs et al., remains one of the most widely validated multimodal scoring systems [8-10]. Multiple iterations of RMI exist, reflecting efforts to refine predictive performance through adjustments to ultrasound criteria and menopausal scoring.

RMI 2, described by Tingulstad et al., modifies the original algorithm by assigning ultrasound and menopausal status scores of 1 or 4 based on the number of morphological features present and menopausal state, respectively [11]. The RMI 2 score is calculated by multiplying the ultrasound score, menopausal score, and CA-125 level, with a threshold of 200 commonly used to differentiate benign from malignant masses. Although RMI 2 offers improved discriminatory power in some populations, its performance may be influenced by operator expertise, tumor subtype, and menopausal status, which can limit consistency across clinical settings [12].

To reduce subjectivity associated with ultrasound interpretation and reliance on tumor markers, the International Ovarian Tumor Analysis (IOTA) group developed standardized sonographic criteria and diagnostic models [13]. The IOTA Simple Rules comprise five benign and five malignant ultrasound features that together provide a structured and reproducible classification system [14]. Large prospective studies have demonstrated high sensitivity and specificity in centers trained in IOTA methodology. Nevertheless, a subset of adnexal masses remains “inconclusive,” requiring expert review or application of more advanced IOTA models such as ADNEX (Assessment of Different NEoplasias in the adneXa) [14,15].

Despite the availability of multiple diagnostic strategies, direct comparisons of IOTA Simple Rules and RMI 2 remain limited, especially in diverse populations and resource-limited environments where differences in operator training, tumor histology, and access to advanced imaging may influence diagnostic performance. Additionally, the optimal approach for managing inconclusive IOTA classifications is not yet fully established.

The primary objective of this study was to compare the diagnostic accuracy of the IOTA Simple Rules and the RMI-2 in distinguishing benign from malignant adnexal masses, using histopathology as the reference standard. Secondary objectives included evaluating the diagnostic performance of serum CA-125 and examining the impact of inconclusive IOTA classifications on clinical interpretation. By evaluating these modalities in a representative cohort, this study aims to inform evidence-based risk stratification strategies for women presenting with adnexal masses and to provide clinically relevant insights for gynaecologic surgeons and multidisciplinary care teams.

## Materials and methods

Study design

This was a prospective diagnostic accuracy study conducted at Meenakshi Mission Hospital & Research Centre, Madurai, India. The study protocol was reviewed and approved by the Institutional Ethics Committee of Meenakshi Mission Hospital & Research Centre. The study was designed and reported in accordance with the STARD (Standards for Reporting of Diagnostic Accuracy Studies) 2015 guidelines [16]. All procedures were performed in accordance with the ethical standards of the institutional research committee and with the 1964 Helsinki declaration. Written informed consent was obtained from all participants prior to inclusion in the study.

Study population

Inclusion criteria were women aged ≥18 years with ultrasonographically detected adnexal masses scheduled for surgical intervention. Patients with incomplete clinical or histopathological data or with prior known ovarian malignancy were excluded.

A consecutive sampling strategy was used, enrolling all eligible women presenting with adnexal masses to our center between December 2016 and June 2017. This approach ensured the inclusion of all patients meeting eligibility criteria during the study period.

Clinical assessment and data collection

Baseline demographic and clinical data, including age, menopausal status, parity, presenting symptoms, and relevant laboratory values, were collected for all participants. Menopausal status was categorized as premenopausal or postmenopausal based on self-reported menstrual history. Parity was defined as nulliparous or parous. Serum CA-125 levels were measured preoperatively using standard immunoassays.

Ultrasonography and risk assessment

All patients underwent transvaginal and/or transabdominal ultrasonography. All ultrasonographic examinations were performed by a gynecologic imaging specialist using a Philips HD5 Ultrasound machine (Koninklijke Philips N.V., Amsterdam, Netherlands). The operator had 10 years of experience in gynecologic ultrasonography. The sonographers were blinded to serum CA-125 levels, clinical suspicion of malignancy, and final histopathological diagnosis at the time of image acquisition. Standardized IOTA Simple Rules protocols were followed for all assessments. The IOTA Simple Rules were applied according to the IOTA Consortium guidelines [13,14], classifying each mass as benign, malignant, or inconclusive.

The RMI-2 was calculated for each patient following the method of Tingulstad et al. [14]. The ultrasound score (U) was assigned a value of 1 when zero or one morphological feature was present (multilocularity, solid areas, bilaterality, ascites, or metastases) and 4 when two or more features were present. Menopausal status (M) was scored as 1 for premenopausal and 4 for postmenopausal women (defined as ≥1 year of amenorrhea or hysterectomy with age >50 years). RMI-2 was computed using the formula:

RMI-2 = U × M × CA-125 (IU/mL)

A cutoff value of 200 was used to distinguish benign from malignant adnexal masses.

Reference Standard

All patients underwent surgical excision of the adnexal mass and were evaluated by single gynecologic pathologists who were blinded to the IOTA and RMI classifications. Histopathological interpretation followed the WHO 2020/2022 classification system for ovarian and adnexal tumors [14,15]. 

Statistical analysis

All analyses were performed in Jamovi version 2.6.44 (https://www.jamovi.org/). Descriptive statistics were computed for all demographic and clinical variables. Continuous variables are reported as mean ± standard deviation (SD) or median with interquartile range (IQR), and categorical variables as frequencies and percentages.

Diagnostic performance metrics such as sensitivity, specificity, positive predictive value (PPV), negative predictive value (NPV), accuracy, and Youden’s index were calculated with corresponding 95% confidence intervals (CIs). Likelihood ratios and chi-square tests were also computed. Receiver operating characteristic (ROC) curves were plotted for IOTA, RMI (as both binary and continuous variables), and CA-125. The area under the ROC curve (AUC) with 95% CI was calculated for each method. Pairwise comparisons of AUCs were performed using the DeLong test. McNemar’s test was used for direct paired comparison of discordant results between IOTA and RMI. Sensitivity analyses were performed for IOTA simple rules by reclassifying all inconclusive cases first as malignant and then as benign, recalculating all diagnostic metrics in each scenario. ROC curves and diagnostic accuracy metrics were computed using the DiagROC and Frequencies modules in Jamovi.

## Results

**Figure 1:**
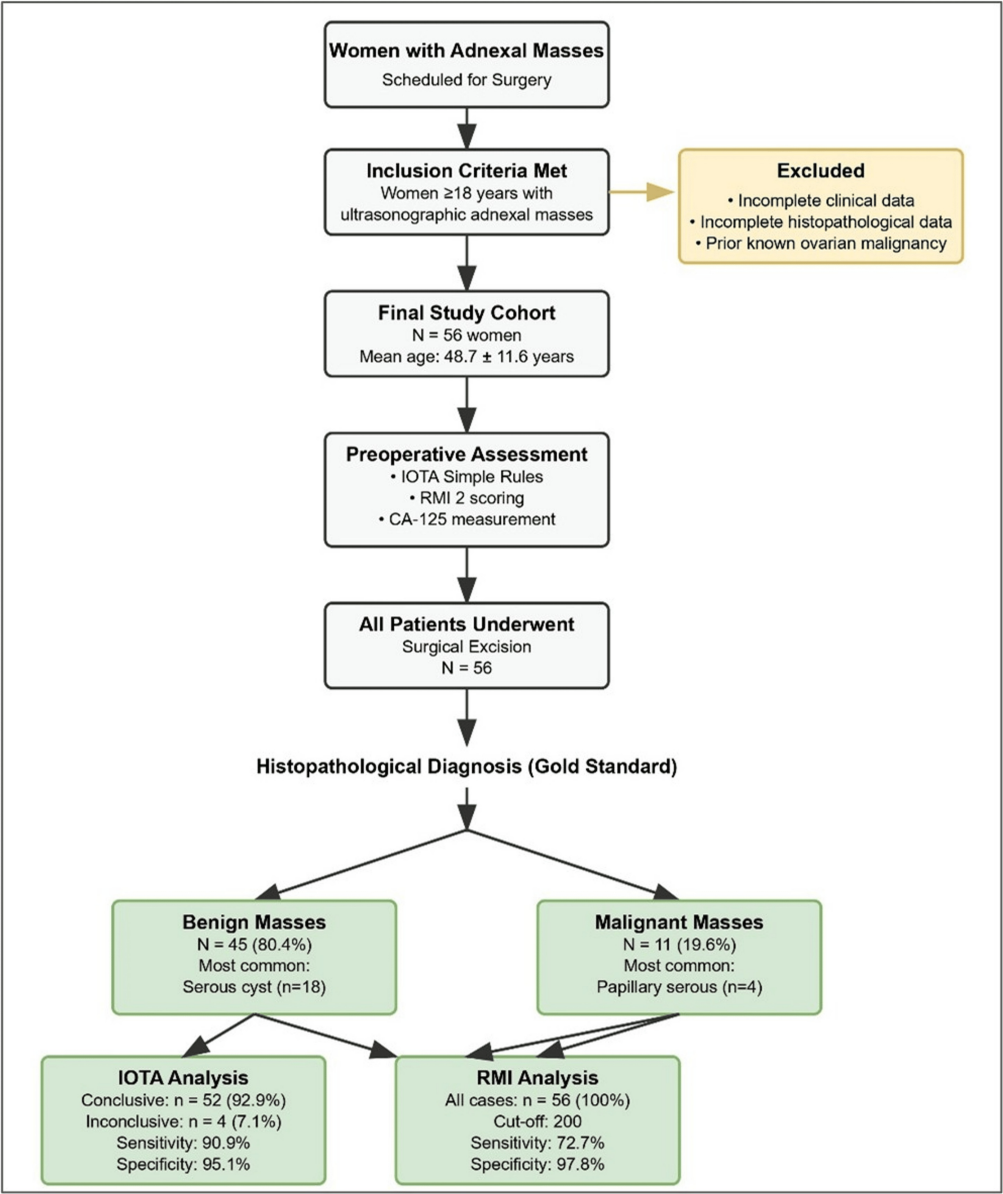
STARD flow diagram showing patient enrollment, exclusions, and classification outcomes. IOTA: International Ovarian Tumor Analysis; RMI: Risk of Malignancy Index; STARD: Standards for Reporting Diagnostic Accuracy Studies

A total of 56 women with adnexal masses were enrolled. The mean age was 48.7 ± 11.6 years (range: 22-78). Of the cohort, 39 (69.6%) were premenopausal and 17 (30.4%) postmenopausal. Nulliparity was noted in 14 (25.0%) patients, while parity was seen in 42 (75.0%). The median CA-125 value was 13.7 U/mL (IQR: 8.7-34.7 U/mL). The median largest mass size was 90 mm (IQR: 60-120 mm) (Figure 1, Table 1).

**Table 1:** Baseline demographic and clinical characteristics of the study cohort (N = 56).

Variable	Value
Age (years), mean±SD	48.7 ± 11.6
Menopausal status, n (%)	
Premenopausal	39 (69.6%)
Postmenopausal	17 (30.4%)
Parity, n (%)	
Nulliparous	14 (25.0%)
Parous	42 (75.0%)
Ca-125 (U/ml), mean±SD	13.7 (8.7–34.7)
Largest mass size (mm), median (IQR)	90 (60–120)

Histopathology findings

Of the 56 cases, 11 (19.6%) were malignant and 45 (80.4%) were benign on final histopathology. The most common benign histology was simple/benign serous cyst (n = 18), followed by benign serous cystadenoma (n = 7) and mature cystic teratoma (n = 6). The most frequent malignant histology was papillary serous cystadenocarcinoma (n = 4), followed by serous cystadenocarcinoma (n = 2) and metastatic adenocarcinoma (n = 2) (See supplementary file).

Diagnostic performance: IOTA and RMI

IOTA Simple Rules (Conclusive Cases)

IOTA Simple Rules were conclusive in 52/56 cases (92.9%) (Table 2). 

**Table 2:** Distribution of benign and malignant cases as classified by IOTA Simple Rules compared to gold standard histopathology (N=52*) *conclusive cases only IOTA: International Ovarian Tumor Analysis

	Histopath benign	Histopath malignant	Total
IOTA benign	39	1	40
IOTA malignant	2	10	12
Total	41	11	52

RMI (Cutoff 200) Analysis

All 56 patients had RMI scores with a cut-off of 200 (Table 3).

**Table 3:** Distribution of benign and malignant cases classified by RMI, compared with histopathological diagnosis (N = 56) RMI: Risk of Malignancy Index

	Histopath benign	Histopath malignant	Total
RMI benign	44	3	47
RMI malignant	1	8	9
Total	45	11	56

 Table 4 shows a comparison of diagnostic metrics as per the IOTA Simple Rules and RMI.

**Table 4:** Comparison of diagnostic metrics for IOTA and RMI. IOTA demonstrated higher sensitivity, while RMI showed marginally higher specificity. PPV: positive predictive value; NPV: negative predictive value; ROC: receiver operating characteristic; AUC: area under the curve; IOTA: International Ovarian Tumor Analysis; RMI: Risk of Malignancy Index

Metric	IOTA Simple Rules (conclusive cases)	RMI (cut-off 200) (all patients)
Sensitivity (%)	90.9 (58.7–99.8)	72.7 (39.0–94.0)
Specificity (%)	95.1 (83.5–99.4)	97.8 (88.2–99.9)
PPV (%)	83.3 (56.1–95.1)	88.9 (52.7–98.3)
NPV (%)	97.5 (85.7–99.6)	93.6 (84.8–97.5)
Accuracy (%)	94.2 (84.1–98.8)	92.9 (82.7–98.0)
AUC (ROC)	0.930 (0.835–1.000)	0.853 (0.713–0.992)
Youden’s index	0.86	0.71
Chi-square (p)	36.2 (<0.001)	–

ROC analysis

**Figure 2:**
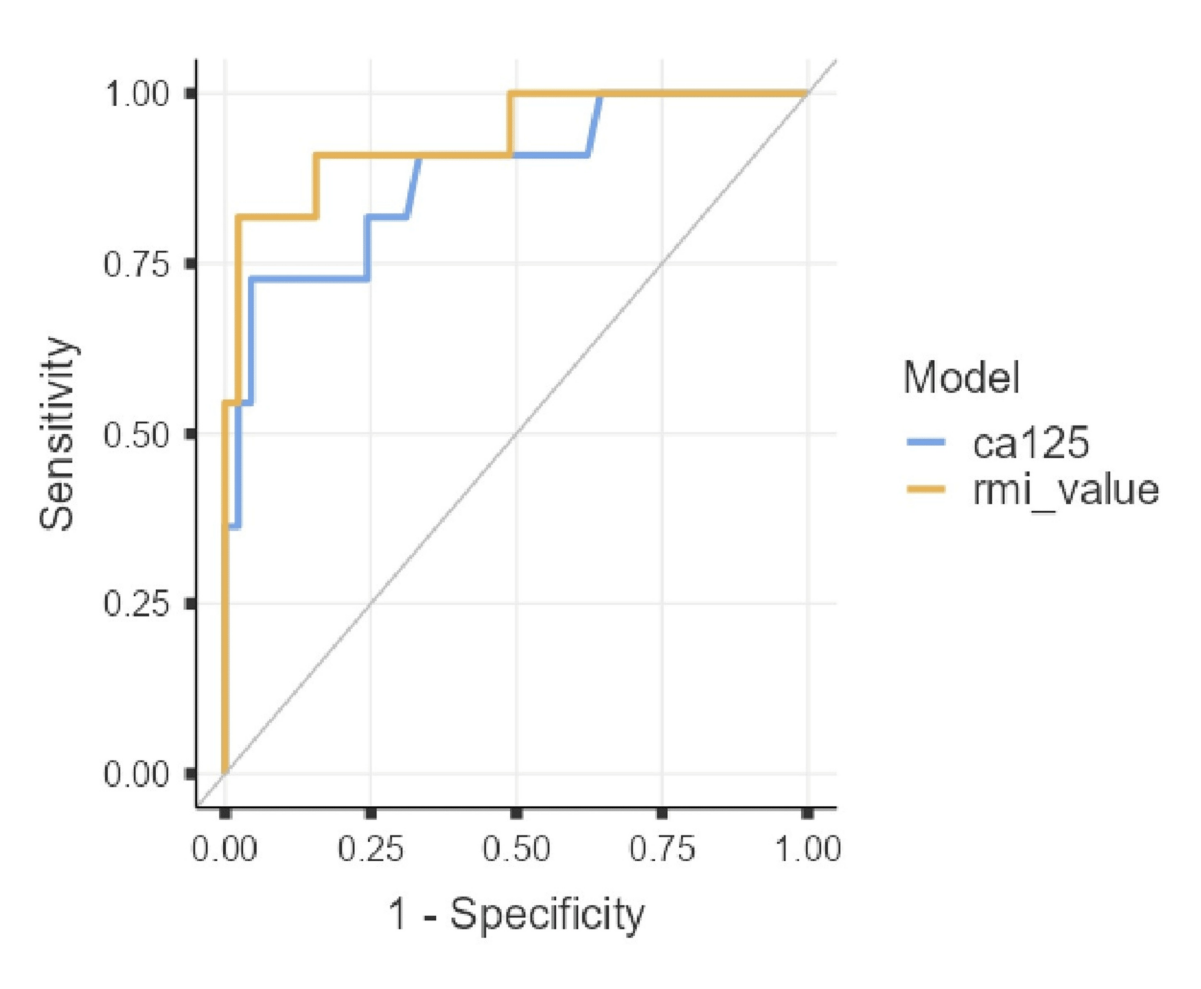
ROC curves for CA-125 (blue) and RMI value (orange) in the diagnosis of ovarian malignancy. The AUC for RMI value was higher, indicating superior diagnostic accuracy compared to CA-125. ROC: receiver operating characteristic; AUC: area under the curve; RMI: Risk of Malignancy Index

**Figure 3:**
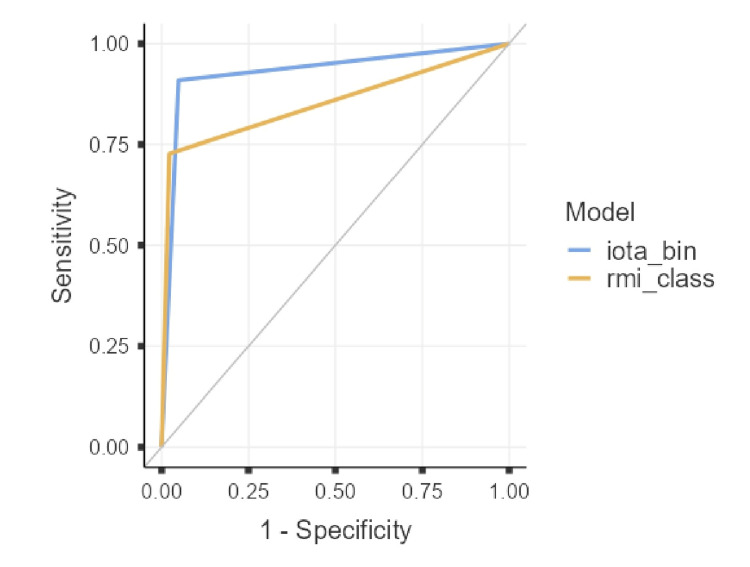
ROC curves for IOTA (blue) and RMI (orange) using standard binary cut-off. IOTA demonstrated the highest AUC, reflecting excellent discrimination between benign and malignant cases. ROC: receiver operating characteristic; AUC: area under the curve; IOTA: International Ovarian Tumor Analysis; RMI: Risk of Malignancy Index

The area under the ROC curve (AUC) for IOTA Simple Rules was 0.930 (95%CI: 0.835-1.000), which was higher than that for RMI (0.853, 95%CI: 0.713-0.992). For continuous RMI value, AUC was 0.935 (0.844-1.00), and for CA-125 alone, 0.879 (0.753-1.00) (Figures 2, 3).

Paired comparison: McNemar’s test

Among the 52 cases with both IOTA and RMI available, McNemar’s test showed no statistically significant discordance (χ² = 3.00, p = 0.083): (IOTA+/RMI: 3 cases, IOTA−/RMI+: 0 cases).

Sensitivity analysis for inconclusive IOTA results

When all inconclusive IOTA results (n = 4) were treated as benign, specificity was 95.6% and PPV 83.3%, with sensitivity unchanged at 90.9%. When all inconclusive results were treated as malignant, specificity fell to 86.7% and PPV to 62.5%, while sensitivity remained 90.9% (Table 5).

**Table 5:** Sensitivity analysis for inconclusive IOTA PPV: positive predictive value; NPV: negative predictive value; IOTA: International Ovarian Tumor Analysis

Scenario	Sensitivity	Specificity	PPV	NPV	Accuracy
Conclusive only	90.9%	95.1%	83.3%	97.5%	94.2%
All inconclusive = benign	90.9%	95.6%	83.3%	97.7%	94.6%
All inconclusive = malignant	90.9%	86.7%	62.5%	97.5%	87.5%

## Discussion

In this prospective diagnostic accuracy study from a tertiary center in South India, we found that IOTA Simple Rules offered excellent discrimination between benign and malignant adnexal masses and outperformed RMI 2 on several metrics. In conclusive IOTA cases (n = 52), sensitivity was 90.9% (10/11) and specificity 95.1% (39/41) with AUC 0.930; by contrast, RMI 2 at the conventional cut-off of 200 (n = 56) yielded 72.7% sensitivity and 97.8% specificity with AUC 0.853. Although AUCs did not differ significantly by DeLong’s test, the pattern of misclassification favored IOTA (fewer false negatives), an attribute that is clinically important for safe preoperative triage. Sensitivity analyses showed that performance remained robust when “inconclusive” IOTA cases were alternatively coded as benign or malignant, underscoring the stability of IOTA’s high NPV in our cohort.

Our results align closely with and extend the contemporary literature [14]. Mongan et al. (Indonesia) observed higher sensitivity and AUC for IOTA compared with RMI in surgical patients (90% vs 65% sensitivity; AUC 0.728 vs 0.689) despite similar specificities, a pattern mirroring our data [17].

Indian investigations corroborate these findings. Abinaya et al. noted superior performance of IOTA-based models over RMI in a prospective hospital cohort, with particularly poor RMI specificity in a benign-predominant sample [15]. Solanki et al. demonstrated IOTA Simple Rules sensitivity of 96.7% and specificity of 92.4% with a very high NPV (99.2%) in 174 operated women [18], which is also consistent with our high NPV and low miss rate for malignancy.

Larger South Asian data further strengthen generalizability. In a 524-patient analysis from Pakistan, IOTA Simple Rules (sensitivity; 94%, specificity; 87%) and ADNEX (AUC ~0.95) outperformed RMI variants, including RMI-2, whose sensitivity hovered near 60% while specificity remained high [19]. More recently, Khastgir et al. prospectively compared Ovarian-Adnexal Reporting and Data System (O-RADS), IOTA Simple Rules, ADNEX, and RMI-4, and found that IOTA Simple Rules and ADNEX achieved the highest overall accuracy (91%), exceeding RMI-4 (88%) and highlighting the consistent strength of IOTA-based approaches across models and settings [20]. Finally, Venkateswaran et al. reported IOTA Simple Rules sensitivity 100% and accuracy 94.7% versus RMI sensitivity 71.4% and accuracy 93.9%, reinforcing the recurring trade-off of higher RMI specificity but materially lower sensitivity and a higher risk of missed cancers [21]. Collectively, these studies across geographies, operators, and case-mix support our conclusion that IOTA sample rules should be the first-line triage tool, with RMI 2 serving as an adjunct where appropriate.

Clinical implications follow directly: in resource-constrained or high-volume services, a method that minimizes false negatives (high sensitivity/NPV) can reduce delayed referral, inappropriate minimally invasive surgery, and suboptimal staging. Our findings, together with the above evidence, argue that wider adoption of IOTA Simple Rules can improve pathways of care, while RMI-2, though specific, should not be relied upon as a sole gatekeeper due to its lower sensitivity.

Although IOTA Simple Rules demonstrated higher sensitivity and a lower false-negative rate compared with RMI-2, the wide confidence intervals, particularly for sensitivity and PPV, reflect the small number of malignant cases and indicate that the study is underpowered to detect statistically significant differences between diagnostic methods. Therefore, our interpretation focuses on the clinical safety profile of IOTA, specifically its lower likelihood of missed malignancies rather than a statistically definitive advantage.

This study has limitations that need to be acknowledged before generalizing the findings. First, it is single-center with a modest malignant case count, which widens CIs and may limit precision for subgroup estimates. Second, a surgical cohort introduces spectrum/verification bias, which is a common constraint in diagnostic studies of adnexal masses. Third, operator dependency inherent to ultrasound could influence test performance despite standardized IOTA application. Fourth, although we defined and implemented RMI-2 rigorously, we did not undertake a head-to-head within-sample comparison against ADNEX, O-RADS, or RMI-4/5, which several recent reports have evaluated [14,19]. Finally, external validation was not performed.

Future work should prioritize multicenter recruitment with larger malignant samples; prospective impact analyses such as change in referral accuracy, surgical staging adequacy, time-to-oncology, and cost-effectiveness; head-to-head comparisons of IOTA Simple Rules, ADNEX, O-RADS, and RMI versions (2/4/5); and inter-/intra-observer reproducibility studies for training and quality assurance. Decision-curve analyses and calibration plots could further clarify clinical utility across prevalence ranges and care levels.

In summary, our prospective evaluation demonstrates that IOTA Simple Rules provide safer and more reliable preoperative triage than RMI-2, consistent with evidence from other studies and multiple Indian cohorts.

## Conclusions

Our study demonstrates that the IOTA Simple Rules offer a clinically valuable and reliable approach for preoperative risk stratification of adnexal masses. Although the study was not powered to establish statistical superiority over RMI-2, the performance profile of IOTA, particularly its high sensitivity and low false-negative rate, indicates a safer clinical pathway for minimizing missed malignancies. Adoption of standardized ultrasound-based algorithms such as IOTA can therefore enhance diagnostic triage, reduce unnecessary interventions, and support improved patient outcomes in gynecologic practice.
